# Quantifying the Impact of Anonymization-Induced Clinical Data Quality Loss: Methodological Quantitative Case Study Using Primary Diagnosis Codes and Hospital Length of Stay

**DOI:** 10.2196/97661

**Published:** 2026-09-11

**Authors:** Gaetan Kamdje Wabo, Piotr Pawel Sokolowski, Mahboubeh Jannesari Ladani, Michael Hagmann, Thomas Ganslandt, Fabian Siegel

**Affiliations:** 1 Department of Biomedical Informatics, Mannheim Institute for Intelligent Systems in Medicine (MIISM), Medical Faculty of Mannheim, University of Heidelberg, Theodor-Kutzer-Ufer 1–3, House 3, Floor 4, Mannheim, 68167, Germany, 49 621 383 8088; 2 Institute of Medical Informatics, Biometry and Epidemiology, Friedrich-Alexander-Universität Erlangen-Nürnberg, Erlangen, Bavaria, Germany

**Keywords:** ADAQI reporting checklist, Anonymization and Data Analysis Quality and Impact Reporting, data anonymization, k-anonymity, clinical data quality, length of stay, International Classification of Diseases, ICD codes, distributional fidelity, inferential reproducibility, linear mixed model, ARX Data Anonymization Tool, medical informatics

## Abstract

**Background:**

The secondary use of electronic health record data requires robust privacy protection. k-Anonymity is widely used to enable data sharing by ensuring that each quasi-identifier combination occurs in at least k records; yet, its analytical impact on clinically meaningful structures remains insufficiently characterized, particularly for the combination of record suppression and microaggregation that arises when a numeric attribute lacks a natural generalization hierarchy. A further gap is that anonymization tools report internal information-loss values but do not signal the downstream distributional and inferential distortions these transformations introduce.

**Objective:**

This study evaluated the analytical footprint of k-anonymity at k=5, 10, and 15 on 2 core data elements in retrospective hospital research: primary *International Classification of Diseases, 10th Revision, German Modification* (ICD-10-GM) diagnosis codes, and hospital length of stay (LOS). It aimed to determine and quantify whether anonymization introduces meaningful distortions not captured by the anonymization tool itself, and whether diagnosis-specific LOS patterns remain reproducible after anonymization.

**Methods:**

We analyzed 719,387 inpatient encounters from University Hospital Mannheim from 2010 to 2024. Anonymization was performed with the ARX tool. It used record suppression and microaggregation. Distributional distortion was assessed with the Kolmogorov-Smirnov *D* statistic, quantile shifts, IQR changes, and tail changes. Categorical fidelity was assessed with the Jaccard coefficient and Cramer *V*. Inferential reproducibility was assessed with a 3-level linear mixed model. The model included random intercepts for the three-character diagnosis codes from the *International Classification of Diseases* (*ICD-3*) and patients. We compared the intraclass correlation coefficient and diagnosis-level effect concordance. Concordance was quantified using Spearman ρ and Lin concordance correlation coefficient, both with 95% CIs. A composite traffic-light verdict summarized the results.

**Results:**

ARX masked quasi-identifier cells rather than deleting rows; the proportion of encounters with a masked cell rose from 0.77% (k=5) to 2.62% (k=15), distributed almost uniformly across admission years. Kolmogorov-Smirnov *D* was stable at 0.147. Median LOS shifted by 1 day, and the SD declined by about 6.5 days, while the diagnosis-level mean changed considerably (absolute mean shift of −1.81 days). Jaccard overlap fell from 0.624 to 0.421. The diagnosis intraclass correlation coefficient rose from 0.294 to 0.837, reflecting variance compression rather than improved signal. Best linear unbiased prediction rank concordance (Spearman ρ 0.964‐0.970) and aggregate magnitude agreement (Lin concordance correlation coefficient 0.959‐0.966) were high; yet, about 7% of low-signal diagnoses showed sign reversals. Most distributional change occurred at k=5.

**Conclusions:**

k-Anonymity preserved the ranking of diagnosis-specific LOS effects but altered distributional shape, individual effect magnitudes, and diagnostic vocabulary, none of which was flagged by the internal loss metric. Anonymized data of this type may support ordinal analyses but can mislead analyses requiring faithful variance structure, accurate absolute effects, or complete rare-diagnosis representation. A reporting checklist is provided to document these effects.

## Introduction

### Background

The increasing use of electronic health record data for secondary purposes has become a cornerstone of clinical research, health services planning, and AI development in medicine [1]. This progress creates an inherent tension. Patient privacy must be safeguarded, while data quality must remain sufficient for valid statistical inference. The European General Data Protection Regulation [2] codifies strict obligations for processing personal health information. Under the General Data Protection Regulation, anonymization may allow broader data sharing where the applied transformations make reidentification sufficiently unlikely in the relevant context.

Among the most widely adopted paradigms, k-anonymity, proposed by Sweeney [3], guarantees that each combination of quasi-identifying attributes appears in at least k records. The ARX Data Anonymization Tool, developed by Prasser et al [4], implements k-anonymity alongside l-diversity [5], t-closeness [6], and differential privacy [7] within a unified open-source framework. ARX applies generalization hierarchies, record suppression, and microaggregation to achieve privacy guarantees while optimizing utility through configurable loss functions [8]. Its application spans clinical trial data sharing [9], pandemic cohort publication [10], and multisite registry studies [11]. Its broad acceptance in the research community is precisely what makes it a suitable target tool for a methodological case study, because findings obtained with ARX are relevant to a large body of existing and future work.

Despite the maturity of anonymization algorithms, systematic evaluation of downstream statistical consequences remains underdeveloped. Five principal approaches currently inform quality assessment. Information loss metrics embedded within tools capture transformation cost but not external analytical validity [4]. Descriptive concordance methods compare summary statistics, with Ohno-Machado et al [12] showing that suppression preserves means while degrading prediction. CI overlap approaches, as demonstrated by Pilgram et al [13], evaluate analytical agreement under use-case–specific configurations. Distributional divergence methods use statistical tests, applied for instance by Jakob et al [10] to COVID-19 anonymization pipelines. Regression reproducibility frameworks, as used by Johann et al [14], test whether clinical associations survive transformation. Recent work continues to confirm that privacy-enhancing transformations degrade analytical utility in ways that depend on the downstream task. Cohen et al [15] quantified the effects of pseudonymization on 6 archetypal epidemiological analyses in a clinical data warehouse and showed that achieving low reidentification risk required modifications that compromised the reliability of specific statistics, and Kaabachi et al [16] cataloged in a scoping review the heterogeneous privacy and utility metrics used across medical studies and the difficulty of identifying an optimal balance.

Each of these approaches illuminates one facet of the privacy-utility trade-off. However, their isolation limits explanatory power. Distributional fidelity metrics quantify what changed in the data structure but cannot determine whether those changes compromise downstream conclusions. Inferential reproducibility tests reveal whether conclusions changed but, without distributional context, cannot explain why or through which transformation mechanism. Combining both dimensions within a single evaluation approach can address this interpretive gap. Distortion patterns such as variance compression or vocabulary erosion, once identified through distributional analysis, can be directly linked to concordance or divergence in the statistical estimates derived from the transformed data. This gap is particularly relevant when anonymization relies on record suppression combined with microaggregation, a configuration that arises whenever generalization hierarchies cannot be specified for all quasi-identifiers. Numeric attributes such as hospital length of stay (LOS) lack a natural semantic hierarchy [17], compelling tools such as ARX to apply microaggregation instead [8]. The analytical consequences of this specific combination remain largely unquantified, as existing evaluations have focused predominantly on generalization-based pipelines [13].

Against this methodological backdrop, 2 clinical data elements occupy a uniquely central position in hospital research. These are the primary diagnosis coded in the *International Classification of Diseases* (ICD) system and the hospital LOS. Primary ICD codes define case classification for diagnosis-related group reimbursement [18], serve as epidemiological cohort criteria, and anchor outcome research. The LOS functions as a proxy for disease severity, resource consumption, and care efficiency [19]. The association between diagnosis and LOS has been extensively investigated [20-29]. Any anonymization-induced distortion of these attributes may directly threaten the validity of downstream analyses. Given this context, 3 research questions (RQs) guided this investigation.

### RQs

The 3 RQs are as follows:

- RQ1 (distributional fidelity): To what extent does k-anonymity at k=5, 10, and 15 alter the distributional properties of primary *International Classification of Diseases, 10th Revision, German Modification* (ICD-10-GM) diagnosis codes and hospital LOS, as measured by descriptive divergence statistics (Kolmogorov-Smirnov [KS] *D*), complementary distributional descriptors (quantile shifts and IQR compression), Cramer *V*, and the Jaccard similarity coefficient?
- RQ2 (inferential reproducibility): Does the diagnosis-associated variation in hospital LOS remain reproducible after anonymization? In clinical terms, if a researcher uses anonymized data to identify which diagnoses are associated with the longest hospital stays, will the resulting ranking agree with the ranking obtained from the original data, and will the estimated magnitude of these differences remain sufficiently stable for interpretation?
- RQ3 (dose-response shape): Does data quality degrade gradually with increasing k, or does most of the change occur at the first anonymization step? The practical implication is whether choosing a higher k level (eg, k=15 instead of k=5) imposes a substantially greater analytical cost, or whether the initial transformation already accounts for the dominant share of the change.

## Methods

### Overview

This methodological case study combines distributional impact assessment with inferential reproducibility testing on a large-scale hospital encounter dataset. The intent is not to model a comprehensive real-world data release, but to demonstrate, using a widely adopted anonymization tool under self-configured anonymity, transformation, and utility parameters, that meaningful distributional and inferential distortions can occur which the tool itself does not report. The analysis proceeds in 4 phases: cohort extraction and characterization, anonymization at 3 k-anonymity thresholds using ARX, distributional distortion quantification through a purpose-built comparison framework, and inferential reproducibility evaluation by fitting identical linear mixed model (LMM) specifications to original and anonymized data.

### Ethical Considerations

This study received approval from Ethics Committee II, Medical Faculty Mannheim, Heidelberg University (ID: 2024‐885). A data protection impact assessment was positively evaluated. The local Use and Access Committee granted formal approval. All analytical datasets were processed and handled in deidentified form within the approved institutional governance framework.

### Data Collection and Cohort Description

For this evaluation, hospital encounter data were extracted from the clinical data warehouse of the Medical Data Integration Center at University Hospital Mannheim (Universitätsmedizin Mannheim). The query targeted inpatient encounters of the study period. For the present analysis, the cohort was rederived directly from recorded admission and discharge dates, retaining only encounters whose admission date and discharge date both fell within January 1, 2010, and September 30, 2024 (revised data preparation in Multimedia Appendix 1). The resulting dataset comprised 5 data elements per encounter: a pseudonymous patient identifier, a unique encounter identifier, the admission year, the primary ICD-10-GM diagnosis code, and the LOS. LOS was computed as the difference in days between discharge and admission, with same-day encounters assigned a value of 1. Because the cohort was defined on the basis of present and valid admission and discharge dates, no encounter had a missing LOS value, and no encounter was excluded for that reason.

### Data Anonymization

Anonymization was performed using the ARX Data Anonymization Tool (version 3.9.2) [4]. Three separate configurations were applied to the same source dataset (N=719,387 encounters), producing anonymized variants at k=5, k=10, and k=15. Secure Hash Algorithm 256-bit checksums verified each output. Table 1 details the complete ARX configuration. Two quasi-identifiers were designated: the primary ICD diagnosis code (categorical) and LOS in days (integer). The patient identifier and admission year were retained outside the quasi-identifier set, the former to enable patient-level random effects and the latter to enable a temporal sensitivity analysis, and neither was anonymized. These 2 variables were not intended for external release.

**Table 1.** Configuration parameters of the ARX Data Anonymization Tool (version 3.9.2) applied to 719,387 inpatient encounters.

Parameter	Setting
Anonymization model	k-Anonymity
k values tested	5, 10, 15
Quasi-identifiers	Primary ICD-10-GM^a^ code, LOS^b^ (days)
Retained non–quasi-identifiers	Patient ID, encounter ID, admission year (not anonymized and not intended for release)
ICD^c^ transformation	No generalization hierarchy (height 1, min=max = 0)
LOS transformation	Microaggregation (geometric mean, ordered)
Microaggregation cluster size	Determined by ARX optimizer per k level
Suppression limit	0.50 (maximum fraction of records suppressible)
Suppression mechanism	Cell-level masking of ICD and LOS jointly (token *)
Optimization weights	ICD=0.50, LOS=0.50
Aggregate loss function	Geometric mean
Attacker models	Prosecutor, journalist, marketer (simultaneous)
Risk thresholds	Calibrated inversely to k
Search strategy	Optimal (deterministic, search space size=1)
Software version	ARX 3.9.2
Verification	SHA-256^d^ checksums per output

a ICD-10-GM: International Classification of Diseases, 10th Revision, German Modification.

b LOS: length of stay (days).

c ICD: International Classification of Diseases.

d SHA-256: Secure Hash Algorithm 256-bit.

### Rationale for the ARX Configuration

Two configuration choices warrant explicit justification because they directly governed the suppression behavior and the resulting distortion. First, no generalization hierarchy was applied to ICD codes, which were treated as a flat categorical attribute by setting the hierarchy height to 1 with minimum equal to maximum equal to 0 [4,8]. This was deliberate. RQ1 measures how anonymization alters the full ICD code distribution at the level at which codes are originally recorded, and RQ2 evaluates whether diagnosis-associated LOS differences remain reproducible at the 3-character level after the transformation. Pregeneralization would have collapsed full codes (eg, E11.40, E11.41, and E11.49) into their parent category (E11) before anonymization, eliminating the variability that the suppression mechanism is intended to act upon. Suppression would then operate on an already-coarsened code set, and the comparison between original and anonymized data would reflect the combined impact of precoarsening and anonymization rather than the impact of the anonymization step itself. Treating each full code as an indivisible unit ensured that suppression remained the only mechanism acting on the categorical structure.

Second, the suppression limit was set to its maximum admissible value of 0.50, equal optimization weights of 0.50 were assigned to both quasi-identifiers, and information loss was aggregated using the geometric mean. The equal weighting reflects the absence of an a priori reason to privilege either the categorical or the numeric quasi-identifier, and it allows suppression and microaggregation to act with comparable intensity on both attributes rather than concentrating distortion on one of them [4,8]. The geometric mean is the aggregation function used by ARX to combine the normalized per-attribute information losses into a single utility score, and it penalizes uneven loss across attributes more strongly than an arithmetic mean would, which is appropriate when no single attribute should be sacrificed to preserve another [8,17]. The permissive suppression limit was chosen specifically to test whether a high admissible suppression budget would translate into aggressive data deletion. The optimization function was active throughout, and the result is informative in itself. Despite a 50% admissible suppression budget, the optimizer satisfied k-anonymity by masking fewer than 1% of encounters at k=5 and fewer than 3% at k=15 (Multimedia Appendices 2-4), which shows that the observed distortion is not an artifact of brute-force deletion under a loose limit but a structural property of the suppression-plus-microaggregation configuration. The search strategy was the deterministic optimal solution, so the search space contained exactly one transformation per k level; because the ICD hierarchy height was fixed at 1 and no other generalization degrees of freedom existed, there were no alternative thresholds for the tool to optimize across, and reporting a single deterministic solution removes a source of run-to-run variability from the comparison.

However, the equivalence classes that failed to reach the k threshold were resolved exclusively through suppression. In ARX, suppression of this kind does not delete records [30]. The tool replaces the affected quasi-identifier values with a missing token and continues to count and aggregate over the dataset until the k threshold is satisfied. For each encounter that could not be placed in a sufficiently large equivalence class, the primary ICD and the LOS were masked jointly, which confirms record-level rather than independent per-attribute masking. For LOS, microaggregation grouped records into clusters and replaced individual values with the cluster centroid. The geometric mean was used as the microaggregation function so that the imputed centroid is consistent with the logarithmic scale on which the length-of-stay outcome is subsequently modeled. For the strictly positive length-of-stay values in this cohort, the geometric mean equals the exponential of the mean of the log-transformed values, which may reduce the positive bias that arithmetic-mean substitution would introduce under a concave transformation. ARX preserves the original data type of the microaggregated attribute, so the geometric-mean centroids were returned as integer-valued length-of-stay days consistent with the discrete scale of the input variable rather than as continuous floating-point values, and all subsequent statistical tests and model fits were performed on this integer-preserving output.

### Design Note on Analytical Circularity and Study Scope

In this study, LOS serves a dual role. It is designated as a quasi-identifier subject to microaggregation by ARX, and it is simultaneously the response variable in the inferential reproducibility analysis (RQ2). This configuration is a deliberate worst-case evaluation design and is central to the aim of the study. Because microaggregation directly transforms the outcome variable, any observed inferential degradation represents an upper bound on the distortion that would occur when the analytical target is not itself part of the quasi-identifier set. We retained this design intentionally, because the purpose of the study is methodological. We use ICD code and LOS to demonstrate that distributional and inferential distortions can occur under self-configured anonymity and utility settings without the anonymization tool reporting them and to argue that effect-assessment findings of the kind reported here should accompany any analysis performed on anonymized data elements. Introducing an additional clinical outcome that is independent of the quasi-identifier set would weaken the worst-case demonstration and is not necessary for this objective, because the contribution of the study is the transparent quantification and reporting of the transformation footprint, not a substantive clinical claim about LOS. Analysts should interpret the inferential results accordingly. Studies in which the analytical outcome variable is not subject to anonymization transformation may experience substantially less inferential disruption than reported here, and the magnitude reported here should therefore be read as a ceiling.

The selection of exactly 2 quasi-identifiers is likewise a deliberate mechanism-focused design rather than an attempt to model a comprehensive real-world data release. ICD codes and LOS represent the 2 most frequent clinical data types requiring anonymization in hospital settings, namely, a high-dimensional categorical field and a right-skewed numerical outcome. Comparable variable pairings have been examined by Ohno-Machado et al [12], who evaluated suppression effects on diagnostic codes and outcomes; by Pilgram et al [13], who assessed anonymization costs using diagnosis and laboratory data; and by Johann et al [14], who quantified utility loss for clinical scoring variables. By restricting the quasi-identifier set to these 2 elements, the study isolates the interplay between suppression and microaggregation while reducing additional sources of distortion from broader anonymization settings. Variables such as age, sex, and admission date were available in the source warehouse but intentionally excluded, because their inclusion would confound mechanism-specific effects with additional generalization or suppression pathways outside the scope of the RQs. The consequence for external validity is addressed explicitly in the Limitations and Future Work section.

### Distributional Impact Assessment

A purpose-built R function, quantify_anonymization_impact(), was developed to systematically compare original and anonymized datasets [31]. The function accepts 2 data frames and optionally 2 column vectors for targeted ad-hoc comparison.

#### Numeric Distributional Analysis

For each shared numeric column, the function computes the 2-sample KS test. The KS *D* statistic evaluates the maximum vertical distance between empirical cumulative distribution functions [32], *D*=sup|*F*0(*x*)–*F*1(*x*)|. In this study, KS *D* is treated exclusively as a descriptive divergence measure quantifying the magnitude of distributional shift. It does not serve as a calibrated clinical effect size. With sample sizes exceeding 700,000, any nontrivial distributional deviation yields a *D* value that is statistically significant, which renders hypothesis-test interpretation uninformative [33-36]. Instead, *D* anchors a relative comparison across k levels, where a stable *D* indicates that incremental k increases do not add distributional perturbation beyond the initial transformation step. The scaled statistic *z*=sqrt(neff)*D is reported alongside *D* to confirm that the divergence is both large and stable in magnitude across levels, and not as a threshold criterion.

To complement the KS *D*, 2 additional distributional descriptors are reported. Quantile shifts capture changes at the median, providing a clinically interpretable reference point. The IQR change documents the compression of central dispersion. Together, these metrics ground the variance-collapse argument in quantities directly meaningful to hospital resource planning and clinical research [19,22]. The KS and the complementary quantitative metrics were applied to hospital LOS, the single numeric data element in the study data.

#### Categorical Analysis

For each shared categorical column, 3 metrics are computed. The Jaccard similarity coefficient *J*(A,B)=|A intersect B|/|A union B| measures label-space erosion, quantifying whether codes survive anonymization [37,38]. Cramer *V*=sqrt(*χ*^2^/(n*min(*r*–1, *c*–1))) quantifies the magnitude of frequency redistribution among retained shared categories [39,40]. The chi-square test underlying Cramer *V* is computed over shared levels only [41], so the effect size reflects distributional shift rather than conflating it with vocabulary loss. The 2 metrics address complementary facets of categorical degradation. Jaccard captures structural completeness, while Cramer *V* captures distributional fidelity within the surviving code set. They are not redundant.

#### Composite Verdict

The composite verdict is an illustrative heuristic layer designed to make the detailed metrics interpretable for decision-makers and applied researchers. Each comparison yields a traffic-light verdict (green, yellow, or red) based on fixed thresholds for the constituent metrics. A green verdict indicates that the anonymized variable remains suitable for analyses that depend on distributional properties, including variance-dependent methods and prediction models. A yellow verdict signals that comparative or ordinal analyses remain feasible, but analyses dependent on distributional shape or absolute magnitudes require caution. A red verdict warns that anonymization-induced distortion may alter substantive conclusions.

Numeric variables are evaluated in 3 dimensions. For distributional divergence, the KS *D* statistic serves as a summary index of overall distributional shift, with values below 0.05 indicating negligible shift, values between 0.05 and 0.10 indicating moderate shift, and values at or above 0.10 indicating substantial shift. These bands are pragmatic screening thresholds calibrated to the present evaluation context, not universal effect-size benchmarks, and the KS *D* is read jointly with the complementary descriptors of median shift and IQR change. For location shift, the absolute mean shift divided by the original SD produces a dimensionless ratio, classified as tiny at or below 0.10, moderate up to 0.30, and large above 0.30. For spread stability, the SD shift relative to the baseline SD captures whether dispersion is preserved, with deviations within ±10% considered stable and larger deviations indicating compression or inflation. A green verdict requires all 3 conditions jointly, namely, *D* below 0.05, normalized mean shift at or below 0.10, and SD change within ±10%. Yellow requires *D* below 0.10 combined with a normalized mean shift at or below 0.30. Every remaining combination triggers red. Categorical variables are evaluated on 3 parallel dimensions. A green verdict requires Cramer *V* below 0.10, Jaccard overlap at or above 0.80, and missing-data drift at or below 5 percentage points. Yellow permits Cramer *V* below 0.30, Jaccard at or above 0.60, and drift at or below 10 percentage points. All other combinations produce red.

#### Threshold Justification

The normalized mean shift thresholds (0.10 and 0.30) align with the standardized effect-size conventions of Cohen for small and medium effects [40]. The SD stability window of ±10% follows analytical precision standards applied in laboratory medicine and biostatistics [42]. The categorical thresholds for Cramer *V* (0.10 and 0.30) correspond to the benchmarks of Cohen for small and medium association effects [40]. The Jaccard thresholds (0.80 and 0.60) were informed by Haizoune et al [43], who applied comparable composite metrics for privacy-utility assessment. The KS *D* screening bands do not derive from a single established convention. They were set to distinguish negligible, moderate, and substantial distributional displacement within this evaluation approach, and their primary function is to enable consistent relative comparison across k levels rather than to serve as absolute cutoffs.

### LMM Specification

#### Overview

An LMM was selected because the analytical goal of RQ2 is not optimal prediction of LOS but a stable, comparable decomposition of LOS variance into a diagnosis-level component and the remaining variation, evaluated under an identical model form across the original and the 3 anonymized datasets. Three considerations motivate this choice. First, the ICD code grouped to 3 characters (ICD-3) is a high-cardinality categorical variable with around 1000 levels [44], which is impractical to estimate as a fixed-effect factor and natural to treat as a grouping variable. Second, a random-intercept specification treats ICD-3 categories as drawn from a common distribution and applies shrinkage, which stabilizes the diagnosis-level estimates that RQ2 compares before and after anonymization [45]. Third, and most importantly for this study, the random-effect variance components and the diagnosis-level best linear unbiased predictions (BLUPs) provide exactly the quantities needed to investigate whether the diagnosis-to-LOS signal is reproducible after anonymization, namely, the intraclass correlation coefficient (ICC) and the per-diagnosis effect estimates. The LMM is therefore the measurement instrument for reproducibility, not a predictive model whose fit is of interest in its own right.

The model was specified as a linear mixed-effects model with crossed random intercepts for ICD-3 category and patient. To address nonindependence of repeated encounters from the same patient, a patient-level random intercept was included alongside the ICD-3 random intercept, while admission year was included as a fixed categorical effect. The outcome was transformed as log(LOS+1), rather than modeled on the raw LOS scale, to stabilize variance and reduce right-skewness in the length-of-stay distribution. Although same-day encounters had already been assigned a LOS value of 1 during data preparation, the same log(LOS+1) transformation was applied consistently across the original and anonymized datasets. Thus, the fitted model was log(LOS+1)=*b*0+*γt*+*uj*+*vp*+*eijpt*, where *b*0 is the fixed intercept, *γt* denotes the fixed categorical effect of admission year *t*, *uj*~*N*(0, σ²ICD-3) is the random intercept for ICD-3 category *j*, *vp*~*N*(0, σ²patient) is the random intercept for patient *p*, and *eijpt*~*N*(0, σ²residual) is the within-encounter residual. Admission year was also retained for year-specific summaries of cell-level ICD and LOS suppression. Estimation used restricted maximum likelihood with the *lme4* package [46,47]. The values of Akaike information criterion (AIC) and Bayesian information criterion were extracted from the restricted maximum likelihood–fitted models.

#### Note on Negative Binomial Regression

A negative binomial generalized linear model was initially considered as an alternative. Fitting MASS::glm.nb() with ICD-3 as a high-cardinality fixed effect across datasets of approximately 700,000 observations did not converge for any dataset, and the dispersion parameter, AIC, and coefficient estimates could not be obtained. This nonconvergence underscores the value of the random-effects approach, which remains computationally tractable regardless of predictor dimensionality.

### Reproducibility Assessment

Inferential reproducibility was operationalized as the degree to which the diagnosis-level signal recovered from anonymized data agrees with the signal recovered from the original data, assessed across 5 complementary quantities that separate variance structure, rank agreement, and magnitude agreement. First, the ICC=σ2_icd3/(σ2_icd3+σ2_patient+σ2_residual) quantifies the proportion of total log-LOS variance attributable to diagnosis-group membership [48], and the Nakagawa-Schielzeth *R*^2^ provides a complementary variance decomposition [49]. Second, rank agreement of the diagnosis-level BLUPs between the original and each anonymized model is assessed with the Spearman rank correlation ρ_S [50], which answers whether diagnoses keep their relative position in the LOS hierarchy. Third, magnitude agreement is assessed with the Lin concordance correlation coefficient (CCC) [51], which evaluates rank preservation and magnitude agreement simultaneously by measuring how closely paired BLUP estimates fall on the identity line, and which decomposes into a precision component (Pearson *r*) and an accuracy component (bias-correction factor *C*b) [51], with interpretive strength-of-agreement bands proposed by McBride [52]. Reporting both ρ_S and CCC follows the principle that a rank correlation can remain high even when absolute magnitudes diverge [53]. Fourth, the fraction of ICD-3 categories whose BLUP changes sign between the original and anonymized model is reported, since a sign reversal indicates a qualitative reinterpretation of the diagnosis-to-LOS relationship, and the median absolute deviation of BLUP pairs provides a magnitude-focused dispersion measure that is resistant to outliers. Fifth, model fit and prediction accuracy are tracked through AIC, Bayesian information criterion, log-likelihood, root mean square error (RMSE), and mean absolute error. To quantify the precision of the 2 agreement statistics, 95% CIs were computed for the Spearman correlation and for the Lin CCC. All analyses were conducted in R (version 4.4.0; R Foundation for Statistical Computing).

## Results

### Cohort Description

The cohort extraction yielded 719,387 inpatient encounters belonging to 370,548 distinct patients, spanning January 2010 through September 2024. LOS ranged from 1 to 557 days, with a mean of 6.52 (SD 9.74) days, and a median of 4 (IQR 2-7) days. Because the cohort was restricted to encounters with valid admission and discharge dates, every encounter had a defined LOS, and no encounter was excluded for missing LOS. The 5 most prevalent primary ICD-10-GM codes were Z38.0 (single live birth, 2.4%), G47.31 (obstructive sleep apnea), C61 (prostate carcinoma), S06.0 (concussion), and I63.4 (cerebral infarction). No single diagnosis exceeded 3%, confirming a diffuse diagnostic landscape typical of tertiary university hospitals. The original dataset contained 7715 distinct full ICD-10-GM codes, mapping to 1404 unique 3-character prefix categories (ICD-3), which served as the diagnosis grouping variable for all mixed-model analyses.

### Anonymization Footprint

ARX satisfied the anonymity criterion at every level with a deterministic search space of size 1 (Multimedia Appendices 2-4) and reported internal information-loss values (geometric mean) of 0.074 at k=5, 0.084 at k=10, and 0.093 at k=15. Consistent with the mechanism described in the Methods section, ARX did not delete any encounters. The number of rows was identical across the original and all 3 anonymized datasets, and the privacy guarantee was achieved by masking quasi-identifier cells with a missing token (data_point_removal.csv in Multimedia Appendix 5). The proportion of encounters carrying at least 1 masked quasi-identifier cell rose from 0.77% at k=5 (5564 encounters) to 1.71% at k=10 (12,315 encounters) and 2.62% at k=15 (18,858 encounters), and in every masked encounter, the primary ICD and the LOS were masked jointly, confirming record-level rather than independent per-attribute masking (Table 2). The masking was therefore small in volume relative to the cohort, even though the admissible suppression budget was 50%, which indicates that the optimizer did not resort to aggressive deletion to reach the privacy threshold.

**Table 2.** Anonymization summary and suppression documentation across k-anonymity thresholds^a^.

Metric	Original	k=5	k=10	k=15
Total records (rows), n	719,387	719,387	719,387	719,387
Encounters removed (rows deleted), n	0	0	0	0
Encounters with ≥1 masked quasi-identifier cell, n (%)	0 (0)	5564 (0.77)	12,315 (1.71)	18,858 (2.62)
Encounters with complete ICD^b^ and LOS^c^, n (%)	719,387 (100)	713,823 (99.23)	707,072 (98.29)	700,529 (97.38)
Distinct full ICD codes, n	7715	4814	3803	3256
Information loss (geometric mean)	—^d^	0.074	0.084	0.093

a k denotes the minimum equivalence-class size required by the k-anonymity model. Suppression in ARX masks quasi-identifier cells with a missing token rather than deleting rows. A total of 719,387 inpatient encounters from University Hospital Mannheim (2010‐2024).

b ICD: International Classification of Diseases.

c LOS: length of stay.

d Not available.

The masking was distributed almost uniformly across the 15 admission years rather than concentrated in particular periods (suppression_by_year.csv in Multimedia Appendix 5). Year-specific masking rates ranged narrowly from 0.70% to 0.87% at k=5, from 1.58% to 1.92% at k=10, and from 2.36% to 2.88% at k=15, with no monotonic trend across the study period. Unadjusted temporal drift in the suppression pattern is therefore minimal, and the diagnosis-level comparisons that follow are not driven by a particular admission year.

### Distributional Fidelity (RQ1)

#### Length of Stay

The KS *D* statistic, interpreted as a descriptive divergence measure, registered 0.147 at k=5, 0.147 at k=10, and 0.147 at k=15 (Table 3). The corresponding scaled statistic *z* was 87.80, 87.81, and 87.59, confirming that the divergence is both large and, importantly, near-constant in magnitude across levels. This near-identity of *D* and *z* across the 3 thresholds indicates that the dominant distributional shift occurs at the first anonymization step, with negligible incremental perturbation at higher k.

**Table 3.** Consolidated distributional and inferential metrics across anonymization levels (N=719,387 encounters, 370,548 patients).

Metric	*D*0^a^	*D*5^b^ (k=5)	*D*10^b^ (k=10)	*D*15^b^ (k=15)
Records (rows), n	719,387	719,387	719,387	719,387
Encounters with complete ICD^c^ and LOS^d^, n	719,387	713,823	707,072	700,529
ICD-3^e^ groups, n	1404	1170	1059	979
Groups not estimable versus *D*0	—^f^	234	345	425
LOS KS *D*^g^ (descriptive)	—	0.147	0.147	0.147
LOS KS *z* (scaled)	—	87.80	87.81	87.59
LOS median (IQR) (days)	4 (2-7)	4 (3-6)	4 (3-6)	4 (3-6)
LOS mean shift (SD) (days)	—	−1.81 (−6.45)	−1.81 (−6.47)	−1.82 (−6.48)
LOS verdict	—	Red	Red	Red
ICD Jaccard	—	0.624	0.493	0.421
ICD Cramer *V*	—	0.062	0.093	0.114
ICD verdict	—	Yellow	Red	Red
σ2_icd3^h^	0.200	0.231	0.228	0.234
σ2_patient^i^	0.081	0.010	0.010	0.010
σ2_residual^j^	0.398	0.038	0.036	0.036
ICC^k^ (diagnosis)	0.294	0.827	0.831	0.837
ICC (patient)	0.120	0.037	0.036	0.035
ICC (diagnosis+patient)	0.414	0.864	0.867	0.873
Intercept b0 (SE)	1.634 (0.013)	1.596 (0.014)	1.598 (0.015)	1.597 (0.016)
BLUP^l^ ρ_S^m^ versus *D*0	—	0.970	0.966	0.964
BLUP ρ_S 95% CI	—	0.963‐0.975	0.959‐0.973	0.956‐0.971
BLUP Lin CCC^n^	—	0.966	0.965	0.959
BLUP CCC 95% CI	—	0.960‐0.971	0.959‐0.970	0.948‐0.967
BLUP Pearson *r* (CCC precision)	—	0.971	0.969	0.964
BLUP CCC bias-correction *C*b (accuracy)	—	0.995	0.995	0.994
BLUP sign reversals (%)	—	6.8	6.7	7.3
Median BLUP ratio (*D*k/*D*0)	—	1.049	1.044	1.049
BLUP pair MAD^o^ (*D*k*−D*0)	—	0.077	0.082	0.084

a *D*0: original dataset.

b *D*5/*D*10/*D*15: k-anonymized variants.

c ICD: International Classification of Diseases.

d LOS: length of stay.

e ICD-3: three-character ICD category.

f Not available.

g KS *D*: Kolmogorov-Smirnov *D* statistic (descriptive divergence).

h σ2_icd3: between-diagnosis variance.

i σ2_patient: between-patient variance.

j σ2_residual: within-encounter residual variance.

k ICC: intraclass correlation coefficient. All ICC values were computed from the full-precision unrounded variance components.

l BLUP: best linear unbiased prediction.

m ρ_S: Spearman rank correlation.

n CCC: concordance correlation coefficient.

o MAD: median absolute deviation.

**Figure 1.**
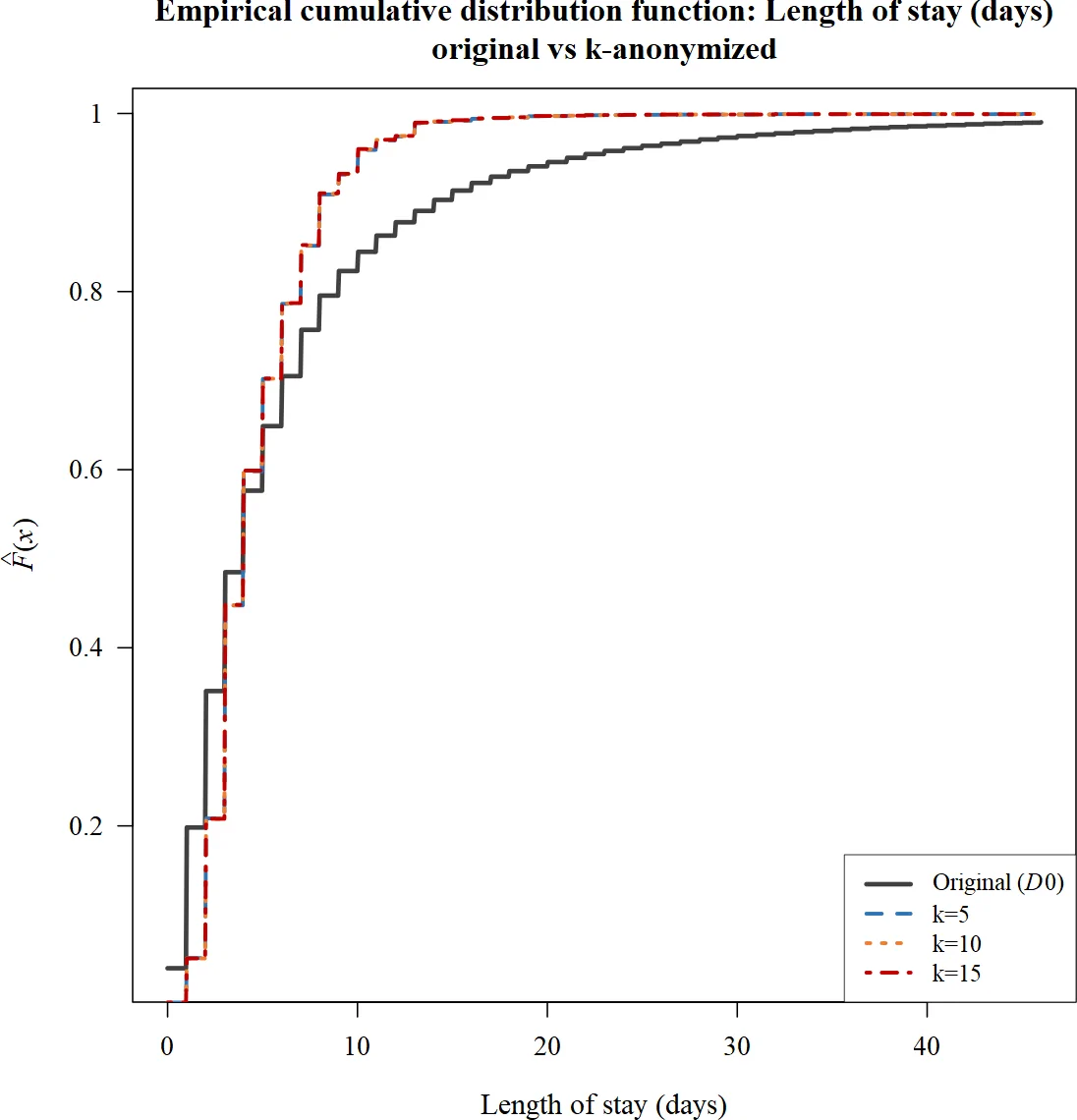
Empirical cumulative distribution functions of hospital length of stay. Black: original dataset (*D*0). Colored: k-anonymized variants. Near-complete overlap among the k=5, k=10, and k=15 curves indicates that the distributional shift saturates at k=5. CDF: cumulative distribution function; LOS: length of stay.

Complementary descriptors characterize the shift. At the distribution level, microaggregation left the median LOS unchanged, from 4 (IQR 2-7) days in the original data to 4 (IQR 3-6) days in every anonymized variant, and narrowed the IQR from 5 to 3 days. This distributional shift, driven by cluster-centroid substitution, is distinct from the cohort-level comparison of masked versus retained encounters reported below, where the median LOS is 4 days in both groups (masked: 4, IQR 2-8; retained: 4, IQR 2-7). The behavior of the statistical moments was asymmetric. The diagnosis-level mean of LOS shifted considerably (absolute mean shift of −1.81 days), whereas the SD compressed by approximately 6.5 days at every threshold (mean shift about −1.81 days and SD shift about −6.45 days on the encounter scale; summary_metrics.csv in Multimedia Appendix 5). Microaggregation replaces within-cluster LOS values with cluster means, which attenuates the second moment far more than the first. An analyst relying on simple location summaries would therefore underestimate the change, while any analysis dependent on variance, CIs, or distributional shape would operate on a materially homogenized outcome. The empirical cumulative distribution function comparison showed that the 3 anonymized LOS curves overlap almost entirely and are displaced from the original curve, reinforcing the saturation pattern (Figure 1). Because suppression and microaggregation were applied jointly, the analysis does not isolate which subset of encounters contributed most strongly to the displacement.

#### ICD Codes

**Figure 2.**
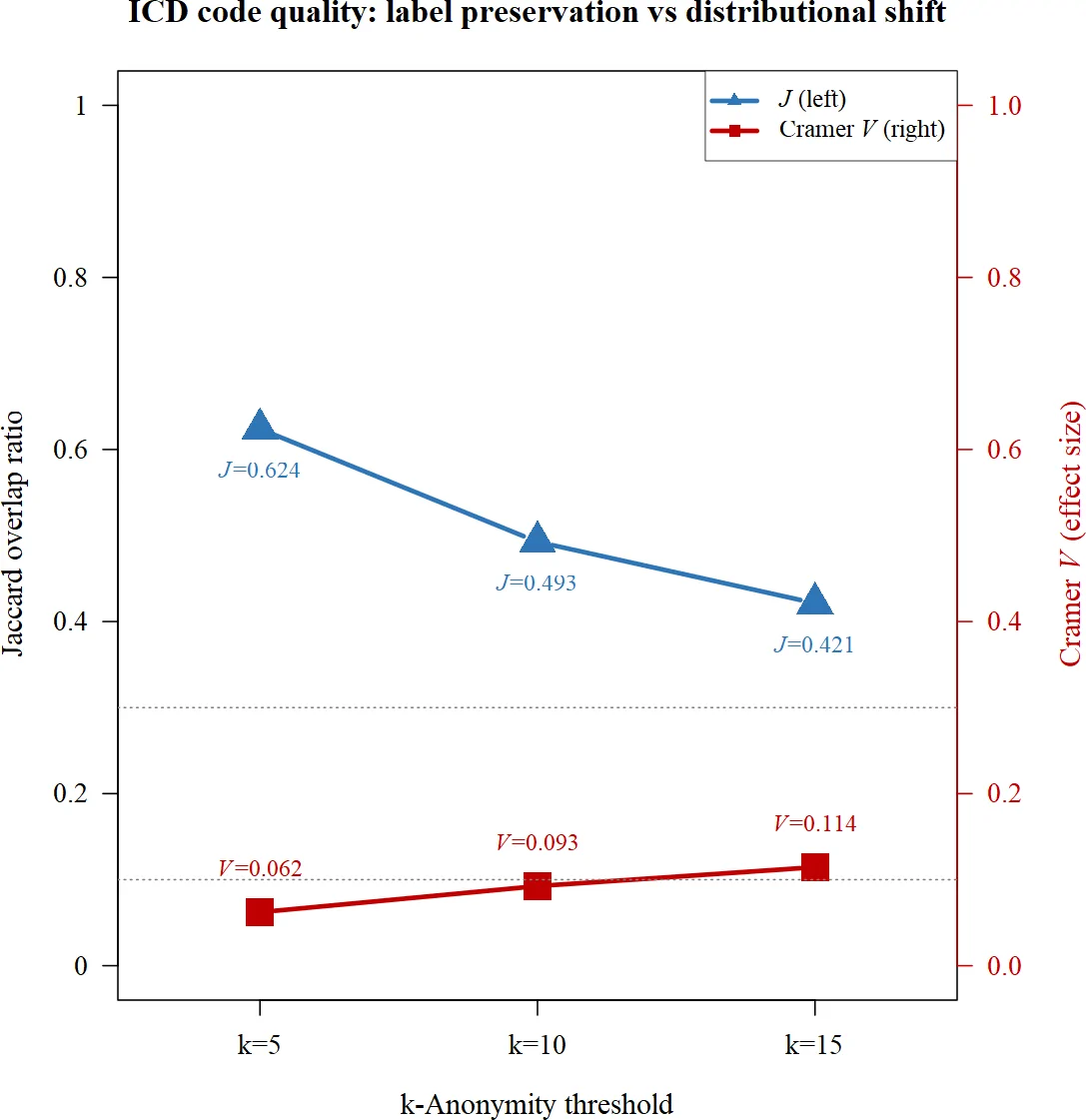
ICD code quality, label preservation versus distributional shift. Left axis: Jaccard overlap ratio quantifying the fraction of original ICD-10-GM codes retained across thresholds. Right axis: Cramer *V* measuring the effect size of frequency redistribution among surviving codes. Horizontal dashed lines mark small (*V*=0.10) and medium (*V*=0.30) effect-size benchmarks. Jaccard captures progressive code-set attrition through suppression of rare diagnoses, while Cramer *V* captures the amplification of association strength as residual frequency mass concentrates on common codes. Both effects intensify monotonically with k. ICD: International Classification of Diseases; ICD-10-GM: International Classification of Diseases, 10th Revision, German Modification.

The Jaccard similarity coefficient declined monotonically from 0.624 at k=5 through 0.493 at k=10 to 0.421 at k=15, reflecting progressive vocabulary erosion. Cramer *V* increased from 0.062 to 0.093 and 0.114. At the strictest threshold, more than half of the original ICD-10-GM codes no longer appeared. Categorical degradation, unlike LOS distortion, exhibited a clear dose-response gradient (Figure 2).

### Inferential Reproducibility (RQ2)

#### Variance Decomposition and ICC

The 3-level LMM fitted to the original data attributed 29.4% of log-LOS variance to ICD-3 category (ICC_icd3=0.294) and a further 12% to the patient level (ICC_patient=0.120), with diagnosis and patient together explaining 41.4% of the variance (Table 3). The between-diagnosis variance (σ2_icd3) was 0.200, the between-patient variance (σ2_patient) was 0.081, and the residual variance (σ2_residual) was 0.398. After anonymization, the ICC attributable to diagnosis rose sharply to 0.827 at k=5, 0.831 at k=10, and 0.837 at k=15. This near-tripling must be interpreted with caution. It is a consequence of microaggregation-induced compression of the residual variance combined with selective masking of atypical records, not an indicator of improved diagnostic explanatory power. Between-diagnosis variance rose moderately from 0.200 to between 0.228 and 0.234, while residual variance collapsed roughly 10-fold from 0.398 to between 0.036 and 0.038, and between-patient variance fell from 0.081 to about 0.010. Because the ICC is a ratio of a between-group variance to the total variance, an operation that disproportionately reduces the denominator inflates the ICC mechanically [48,54-57]. Elevated ICC values in anonymized data should therefore be read as a warning signal of compressed outcome variability, not as evidence that diagnosis categories explain clinical LOS variation more effectively. The number of ICD-3 groups represented in the model decreased from 1404 in the original data to 1170 at k=5, 1059 at k=10, and 979 at k=15, corresponding to 234, 345, and 425 categories no longer estimable after anonymization.

#### Between-Diagnosis Variance

The moderate rise in between-diagnosis variance was analyzed (variance_inflation_interrogation.csv in Multimedia Appendix 5). Between-diagnosis variance increased by 15.5% at k=5, 14.1% at k=10, and 17% at k=15 relative to the original value. Over the same range, the number of ICD-3 groups fell from 1404 to 1170, 1059, and 979, and the median group size grew from 91 encounters to 148, 174, and 203. The mechanism is consistent across these quantities. Suppression preferentially removes small, low-frequency diagnosis groups, so the surviving groups are larger, more homogeneous internally, and more separated from one another after microaggregation has collapsed within-group variation. The between-group variance therefore inflates not because diagnoses became more informative, but because the surviving set of diagnoses is sparser and more widely spaced once the rare groups and the within-group spread have been removed.

#### Model Diagnostics

Residual inspection on the log scale showed acceptable symmetry. No patterns threatened the group-level comparison (Fig_QQ_residuals_D0 through Fig_QQ_residuals_D15 in Multimedia Appendix 5). Quantile-quantile plots of the ICD-3 BLUPs confirmed approximate normality of the diagnosis random-intercept distribution. This held across all 4 datasets (Fig_QQ_BLUPs_D0 through Fig_QQ_BLUPs_D15 in Multimedia Appendix 5). The Shapiro-Wilk test on 5000 subsampled residuals (seed=42) indicated significant departures from normality in every dataset. This is expected at N exceeding 700,000. At this scale, the central limit theorem ensures robust variance-component estimation regardless of moderate distributional deviation [44]. All 4 models converged without warnings or boundary estimates (final_analysis.R in Multimedia Appendix 1).

#### BLUP Concordance, Rank Order, and Magnitude

**Figure 3.**
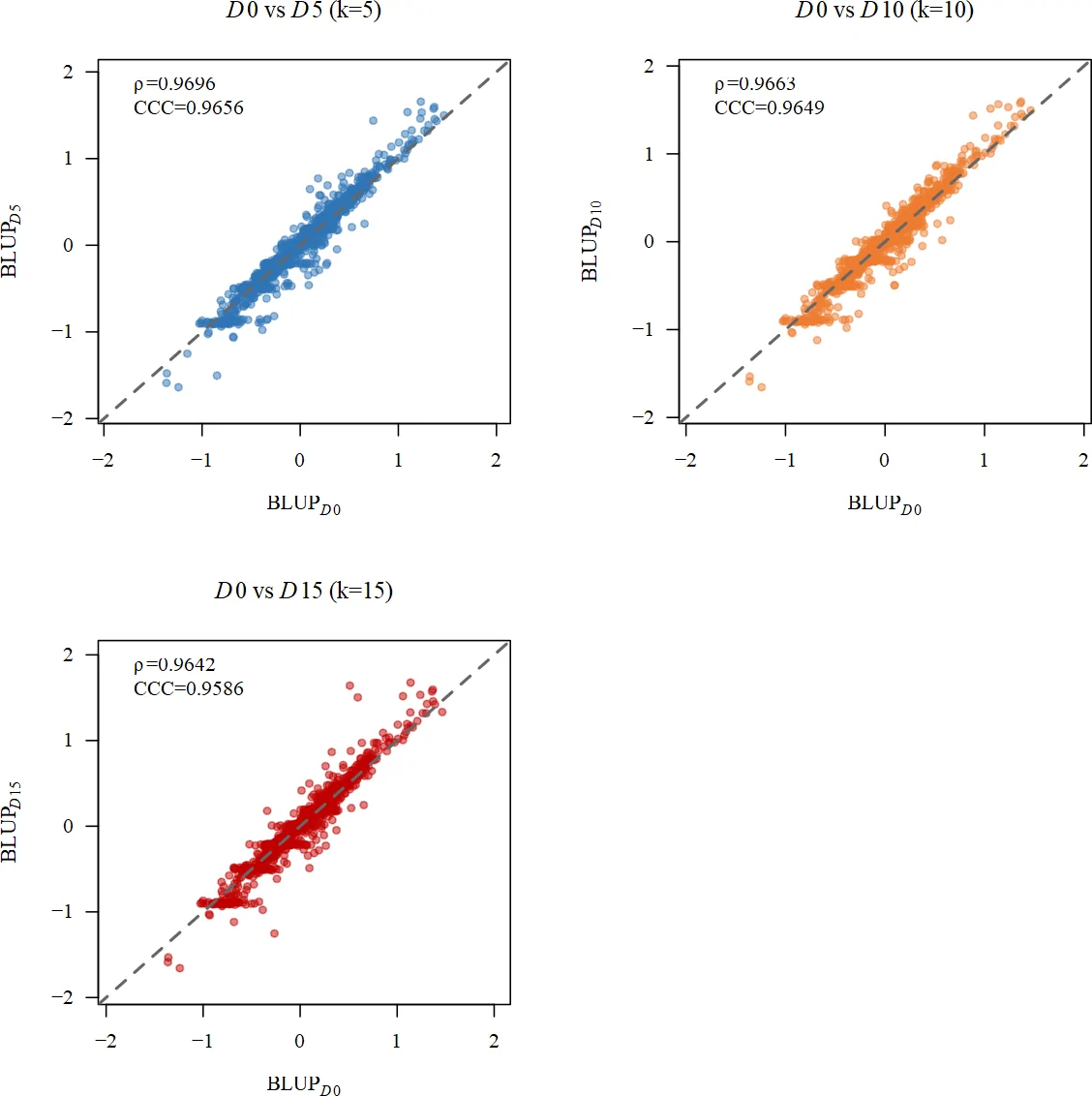
BLUP concordance scatter plots, original versus anonymized data. Each point is one shared ICD-3 category. The Spearman rank correlation is annotated per panel, and the diagonal marks perfect concordance. BLUP: best linear unbiased prediction; CCC: concordance correlation coefficient; ICD-3: three-character ICD category.

Spearman ρ confirmed preserved rank ordering of diagnosis-level effects, at 0.970 (95% CI 0.963-0.975) at k=5, 0.966 (95% CI 0.959-0.973) at k=10, and 0.964 (95% CI 0.956-0.971) at k=15, all with *P* values below machine precision (Figure 3 and BLUP_concordance.csv in Multimedia Appendix 5). A researcher asking which diagnoses carry the longest stays would, in this case study, arrive at concordant rankings from anonymized data. Lin CCC, which integrates precision and accuracy, was also high, at 0.966 (95% CI 0.960-0.971) at k=5, 0.965 (95% CI 0.959-0.970) at k=10, and 0.959 (95% CI 0.948-0.967) at k=15, with Pearson *r* between 0.964 and 0.971. The CIs are narrow, which reflects the large number of shared diagnosis groups on which the agreement statistics are computed (1,170, 1,059, and 979 shared groups). The median BLUP attenuation ratio was close to unity (about 1.04 to 1.05), indicating that the typical diagnosis-level effect was reproduced with little systematic shrinkage or amplification. Decomposing the Lin CCC into its precision and accuracy components confirmed that the high agreement was not a coincidence of offsetting errors. The bias-correction factor *C*b, which isolates the accuracy component, was 0.995, 0.995, and 0.994 across the 3 levels, so the paired estimates lay almost exactly on the identity line rather than on a shifted or rescaled line, and the CCC was therefore driven by precision (Pearson *r*) rather than by chance alignment. The median absolute deviation of the BLUP pairs, a magnitude-focused dispersion measure resistant to outliers, was small and rose only gradually with k, at 0.077, 0.082, and 0.084 on the log scale.

#### Extreme Individual Distortions and Sign Reversals

High aggregate concordance coexisted with extreme distortion of a minority of individual diagnoses, which a single correlation coefficient would conceal (BLUP_extreme_distortion.csv and Fig_extreme_distortion in Multimedia Appendix 5). The diagnoses affected most severely were, almost without exception, those whose original effect was already close to 0, where a small absolute change produces a large relative change or a sign flip. For example, prostate carcinoma (C61) had an original BLUP of only 0.004 and an anonymized BLUP of −0.218 at k=5, which registers as a sign reversal and a nominal ratio far outside the plausible range; nasal-cavity and middle-ear cancers (C30 and C31) behaved similarly. By contrast, diagnoses with substantial original effects were reproduced closely, for example, A02 was reproduced at about 64% of its original value and Z38 at about 77%. Across the shared groups, BLUP sign reversals occurred in approximately 7% of ICD-3 categories at each k level (k=5: 80/1170, k=10: 71/1059, and k=15: 71/979). Moreover, the reversed categories were overwhelmingly those with original effects near 0 on the log scale, where stochastic fluctuation dominates the estimate. This indicates that rank ordering and aggregate magnitude are preserved, while the effect estimate for any specific low-signal diagnosis can be unreliable after anonymization. Meta-analyses pooling anonymized and original estimates for such diagnoses could therefore produce biased summaries despite concordant overall ranking.

#### Prediction Accuracy

As a secondary diagnostic of the variance-compression effect, prediction accuracy was computed across datasets (Table 4). RMSE on the day scale fell from 8.57 in the original data to about 1.58 at k=5 and 1.54 at k=15, and the AIC moved from 1,496,114 in the original data to large negative values (about −161,308 at k=5 to −203,119 at k=15). This apparent improvement does not reflect superior model quality. It is a direct consequence of fitting the model to a variance-depleted response, where the outcome has been compressed toward cluster means. The marginal *R*^2^, which captures only the variance explained by the admission-year fixed effect, was near 0 in every dataset (0.005 in the original data and 0.000 after anonymization), confirming that admission year carried almost no explanatory weight and that the conditional *R*^2^ is driven entirely by the random effects. The rise in conditional *R*^2^ from 0.417 to above 0.86 therefore tracks the same residual-variance compression seen in the ICC rather than any genuine gain in explanatory power.

**Table 4.** Model fit and prediction accuracy across anonymization levels^a^.

Metric	*D*0	*D*5	*D*10	*D*15
AIC^b^	1,496,114	–161,308	–188,380	–203,119
BIC^c^	1,496,320	–161,101	–188,173	–202,912
Log-likelihood	–748,039	80,672	94,208	101,577
RMSE^d^ (log)	0.589	0.179	0.176	0.173
MAE^e^ (log)	0.436	0.108	0.105	0.103
RMSE (days)	8.571	1.581	1.549	1.535
MAE (days)	3.516	0.656	0.639	0.628
Marginal *R*^2^	0.005	0.000	0.000	0.000
Conditional *R*^2^	0.417	0.864	0.867	0.873

a Negative AIC values and reduced RMSE in anonymized datasets reflect a variance-depleted response, not improved model quality (see the Discussion section). Marginal *R*2 reflects variance explained by the fixed effect (admission year) alone; conditional *R*2 additionally includes the diagnosis and patient random effects (Nakagawa-Schielzeth).

b AIC: Akaike information criterion.

c BIC: Bayesian information criterion.

d RMSE: root mean square error.

e MAE: mean absolute error.

### Composite Fitness-for-Purpose Verdict

To make the detailed metrics directly usable by decision-makers and applied researchers, the per-data-element results were summarized with the composite traffic-light verdict defined in the Methods section (Table 5). For LOS, the verdict was red at all 3 thresholds because the SD changed far beyond the ±10% stability window even though the location shift was comparatively small, which is the signature of microaggregation. For ICD vocabulary, the verdict was yellow at k=5, where Jaccard overlap remained above 0.60, and red at k=10 and k=15, where vocabulary loss and frequency redistribution crossed the red thresholds. The verdict therefore communicates a consistent message. The anonymized data of this type support ordinal and comparative use of the diagnosis-to-LOS relationship but are not suitable for analyses that depend on faithful variance structure, accurate absolute LOS estimates, or complete diagnostic vocabulary.

**Table 5.** Composite traffic-light fitness-for-purpose verdict per quasi-identifier and k level, with the governing metric values.

Element	Values, k	Statistic	Mean shift (SD change) (days)	Cramer *V*	Verdict^a^
LOS^b^	5	KS^c^ *D*=0.147	–1.81 (–6.45)	—^d^	Red
LOS	10	KS *D*=0.147	–1.81 (–6.47)	—	Red
LOS	15	KS *D*=0.147	–1.82 (–6.48)	—	Red
ICD^e^	5	Jaccard=0.624	—	0.062	Yellow
ICD	10	Jaccard=0.493	—	0.093	Red
ICD	15	Jaccard=0.421	—	0.114	Red

a A red verdict warns that anonymization-induced distortion may alter substantive conclusions; yellow indicates that ordinal or comparative analyses remain feasible with caution.

b LOS: length of stay.

c KS: Kolmogorov-Smirnov*.*

d Not applicable.

e ICD: International Classification of Diseases.

### Dose-Response Shape (RQ3)

**Figure 4.**
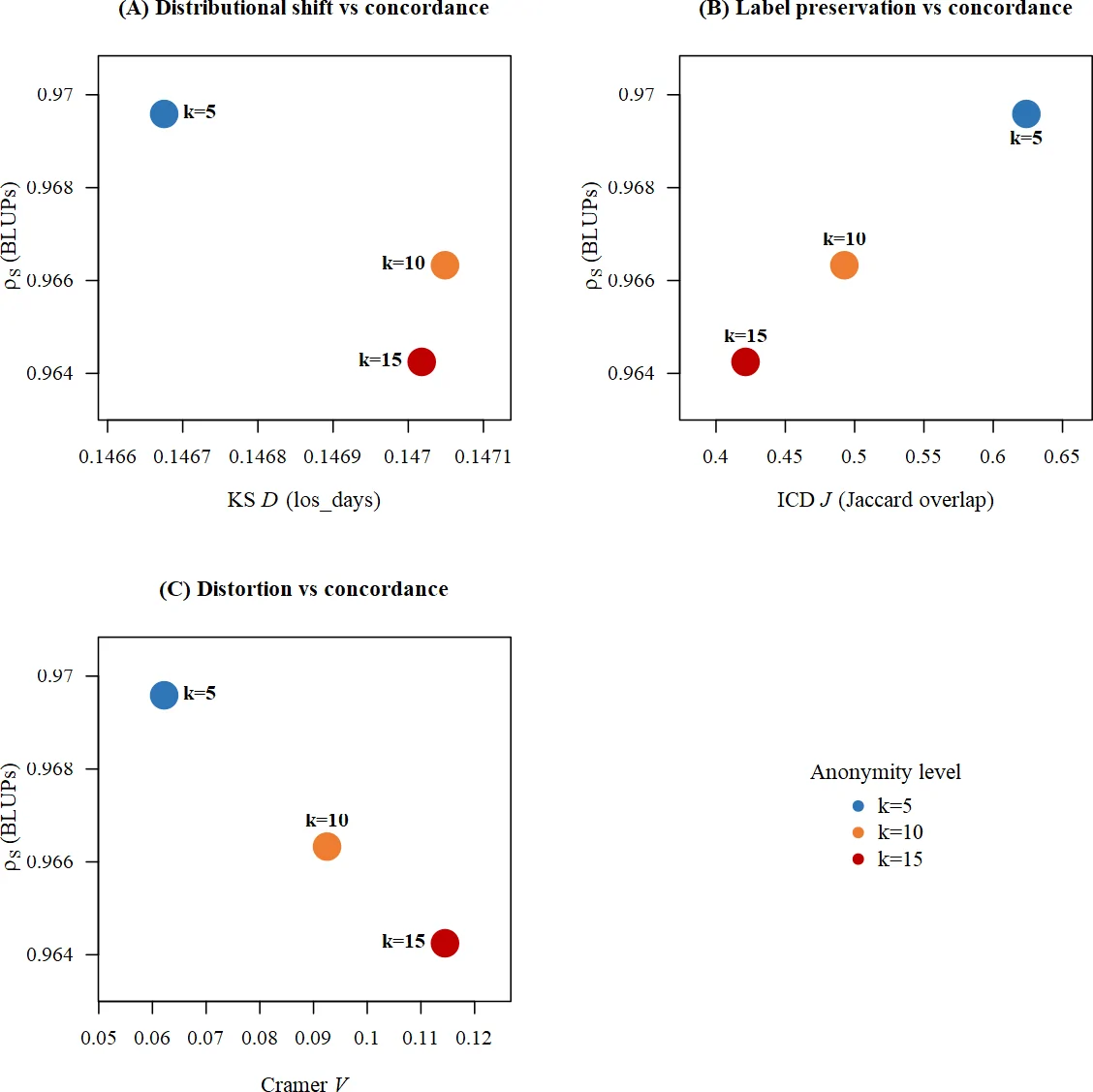
Corroboration of distributional and inferential metrics across k. (A) KS *D* for LOS versus Spearman ρ of BLUPs, showing minimal variation in distributional shift alongside high concordance. (B) ICD Jaccard versus ρ. (C) Cramer *V* versus ρ. The figure shows that increasing k alters distributional and categorical properties more than it alters the reproducibility of relative inferential patterns. BLUP: best linear unbiased prediction; ICD: International Classification of Diseases; KS: Kolmogorov-Smirnov; LOS: length of stay.

Turning to the third RQ, the relationship between k and data-quality change followed a 2-component pattern rather than a single monotonic gradient. For LOS, the KS *D* and its scaled statistic, and all complementary descriptors, were essentially constant across the 3 k levels (Table 3) because microaggregation imposes its distributional footprint at the initial step and additional k increases contribute little further perturbation. For the inferential metrics, the same saturation held. The ICC attributable to diagnosis changed only marginally beyond k=5 (0.827, 0.831, and 0.837), BLUP rank concordance was stable (ρ_S 0.970, 0.966, and 0.964), and Lin CCC changed little (0.966, 0.965, and 0.959). Categorical degradation, by contrast, followed a clear dose-response gradient. Jaccard fell from 0.624 to 0.493 to 0.421, Cramer *V* rose from 0.062 to 0.093 to 0.114, and the number of diagnosis groups no longer estimable scaled with k (234, 345, and 425). The overall pattern is therefore a step-function for the numeric outcome, saturating at k=5, combined with a proportional gradient for categorical erosion, which tracks the suppression of rare codes (Figure 4). This pattern is a property of the present low-dimensional, 2-quasi-identifier configuration and should not be assumed to hold for higher-dimensional releases, as discussed in the Limitations and Future Work section.

However, the KS *D* axis in Figure 4A spans an extremely narrow range because the 3 anonymized LOS distributions are almost identical to one another (KS *D*=0.1467, 0.1470, 0.1470). The points therefore do not order themselves along that axis in a visually monotonic way, and this is expected rather than a sign of instability in the method or the metric. Microaggregation replaces LOS values with cluster means at the first anonymization step, which fixes the shape of the anonymized LOS distribution almost immediately, so that further increases in k change the LOS distribution only negligibly. The near-vertical arrangement of the 3 points along the KS *D* axis is the visual effect of this saturation. The Spearman axis, by contrast, varies smoothly and monotonically because rank concordance is sensitive to the progressive loss of diagnosis groups, which does scale with k. Read together, the 2 axes convey the central finding of the dose-response analysis, namely, that the numeric distortion saturates while the categorical erosion accumulates.

## Discussion

### Principal Findings

This case study produced 5 central observations. First, the distributional comparison identified a substantial LOS perturbation (KS *D*=0.147, SD compression of approximately 6.5 days, contraction of the upper tail) that the internal loss metric of the anonymization tool did not signal, since that metric reported aggregate geometric-mean losses below 10%. In clinical terms, anonymization shifted the median hospital stay from 4 to 5 days and narrowed the IQR from 5 to 4 days, while the diagnosis-level mean changed only modestly. This combination of a small location change with a large dispersion change would remain invisible to any evaluation relying on first-moment comparison alone, which is the core methodological point of the study.

Second, the 2 quasi-identifiers showed mechanism-specific degradation patterns. LOS distortion saturated at k=5, whereas ICD vocabulary loss continued to increase with k. These contrasting patterns are compatible with different dominant transformation mechanisms, but because ARX applies both transformations jointly, the present design supports indirect rather than formal attribution and motivates a future factorial decomposition. Third, microaggregation produced an asymmetric moment pattern. The first moment was largely preserved, while the second moment collapsed, and the upper tail was truncated, which is the expected behavior of cluster-centroid substitution applied to a right-skewed distribution. A small number of long-stay patients drives most of the variance and very little of the mean, so collapsing within-cluster variability removes those tail values almost entirely without shifting the typical value. The practical concern is therefore user-side misinterpretation rather than statistical surprise. An analyst comparing only central tendency would conclude that the data are unaffected, while any variance-dependent analysis would silently operate on a homogenized outcome.

Fourth, the inflation of the ICC after anonymization is most plausibly explained by residual-variance deflation and structural simplification, and it should not be interpreted as evidence of stronger diagnosis-associated information. The interrogation of the variance components showed that the moderate rise in between-diagnosis variance accompanies a sharp fall in the number of diagnosis groups and a near-doubling of median group size, which is consistent with the removal of small, low-frequency groups rather than with any genuine sharpening of the diagnosis-to-LOS signal. Fifth, BLUP rank ordering and aggregate magnitude agreement were both high (ρ_S 0.964 to 0.970, Lin CCC 0.959 to 0.966, each with narrow CIs). However, approximately 7% of diagnosis categories underwent sign reversals, concentrated almost entirely among diagnoses whose original effect was near 0. The headline message of reproducibility is therefore conditional. Relative ranking is robust, but the effect estimate for any specific low-signal diagnosis can be unreliable.

### Characteristics of the Suppressed Encounters

Building on these findings, and because suppression in k-anonymity targets records that fail to reach the k threshold, the masked encounters are not a random sample, and their characteristics were examined directly to assess potential survivor-type bias (analysis of suppressed records.R in Multimedia Appendix 1 and gradual_loss_cascade.csv and cohort_characterization.csv in Multimedia Appendix 5). Masking was small in volume, affecting under 1% of encounters at the first step and under 3% cumulatively at k=15, but it was nonrandom. It fell mainly on lower-frequency codes, with the largest within-code erosion among musculoskeletal diagnoses (eg, M85 lost 43% of its records, M72 lost 28%, and M65 lost 27%), spinal-cord and related injury (G82 lost 33%), trauma codes (S36 and M84), and one rare infection (A31, with 78% of its records removed at k=15). The masked encounters also had modestly longer LOS than those retained, with mean LOS values of 7.23 (SD 13.08), 7.39 (SD 12.58), and 7.38 (SD 12.21) days for k=5, 10, and 15, respectively, compared with 6.49 (SD 9.65), 6.48 (SD 9.62), and 6.47 (SD 9.60) days among retained encounters and 6.52 (SD 9.74) days overall. However, the median LOS remained 4 days in both groups. Because masking operates at the encounter level rather than the patient level, encounter counts reconcile exactly between the suppressed and retained groups, whereas patient counts do not sum to the cohort total, because a single patient may contribute one encounter that is retained and another that is suppressed and therefore appears in both groups (cohort_characterization.csv in Multimedia Appendix 5). This outlier-directed, rare code–directed pattern explains the survivor-type signal seen elsewhere in the results, and it is reported here as a limitation. Estimates for the specific rare or long-stay conditions most affected by masking warrant particular caution, while the aggregate distributional and rank-level results remain largely unaffected because the masked volume is small.

### Clinical and Ethical Implications of Vocabulary Erosion

Beyond the statistical consequences already described, the progressive loss of diagnostic vocabulary carries clinical and ethical implications. At k=15, more than 400 three-character diagnosis categories were no longer estimable, and the analysis above shows that the categories most completely erased are precisely the rare and lower-frequency conditions. Systematically removing rare diagnoses from a research dataset risks rendering those conditions invisible to exactly the secondary analyses that are meant to serve them, such as health-services planning, capacity forecasting, and equity monitoring for uncommon or resource-intensive conditions. If anonymized administrative data are used to allocate resources or to define cohorts, the suppression of rare-disease codes can bias planning toward common conditions and against the long tail of rarer presentations, with the affected patient groups least able to absorb the resulting underrepresentation. This constitutes a fitness-for-purpose concern rather than a privacy concern, and it reinforces the argument that the suppression footprint, and specifically which clinical categories were erased, should be reported alongside any analysis of anonymized clinical data.

### Implications for Researchers

Taken together, for medical informaticians and data scientists, these results carry practical consequences. Mixed-model estimates from anonymized data of this type may appear misleadingly precise, with elevated explained-variance values and narrower prediction intervals that mask true patient-level variability. The roughly 10-fold residual-variance compression means that CIs derived from the anonymized data are narrower than warranted, which can inflate apparent statistical significance through a mechanism analogous to range restriction in psychometric research [54]. The inflated ICC does not mean that diagnoses explain LOS better after anonymization, only that less unexplained variability remains for them to compete against. Researchers identifying which diagnoses carry the longest or shortest stays would, in this case study, reach concordant rankings from anonymized data, but analyses requiring precise effect magnitudes, such as cost projections, resource-allocation models, or meta-analytic contributions, would be compromised by the magnitude distortion and the low-signal sign reversals documented earlier. The apparent improvement in prediction accuracy should be read in the same light. The lower RMSE on anonymized data reflects a compressed outcome, not a better model, and any prediction metric reported on anonymized data should be accompanied by the corresponding metric on the original data and by the variance ratio of the outcome.

### Contextualization Within the Literature

These observations can be situated within the existing literature. Pilgram et al [13] evaluated anonymization costs in a chronic kidney disease cohort and reported that use case–specific configurations yielded higher reproducibility than generic approaches. The present study extends this by showing that even a generic configuration preserves rank-order inferential structure when evaluated through BLUP concordance, while magnitude fidelity is high in aggregate but fails for individual low-signal diagnoses. Johann et al [14] compared anonymized and synthetic heart-failure data and concluded that both degraded utility, which the present granular distributional and quantile-level characterization corroborates and refines. Jakob et al [10] evaluated a COVID-19 anonymization pipeline using distributional comparisons but did not test whether clinical associations survived the transformation, a gap addressed here by the mixed-model reproducibility layer. Ohno-Machado et al [12] showed that cell suppression preserves descriptive statistics while degrading predictive performance, which the present study sharpens by demonstrating that the diagnosis-level mean shifts modestly while the distributional structure changes substantially. The broader contemporary literature aligns with these findings. Cohen et al [15] showed in a clinical data warehouse that reducing reidentification risk required transformations that compromised the reliability of specific epidemiological statistics, and Kaabachi et al [16] documented the absence of a single agreed privacy-utility metric set across medical studies. The present contribution is complementary, namely, a transparent, tool-agnostic procedure for documenting which analytical properties a given anonymization configuration preserves and which it does not, and a reporting checklist to make that documentation routine. Kohlmayer et al [17] focused on information-theoretic loss functions internal to ARX, and the external evaluation performed here reveals quality dimensions that those internal metrics cannot capture. As with the tool-agnostic anonymity verification of pyCANON [58] and related independent utility-assessment approaches [59,60], fitness-for-purpose evaluation should be independent of the anonymization software used. Finally, the dual-metric strategy of supplementing Spearman correlation with Lin CCC operationalizes the principle of Bland and Altman [53] that correlation alone is insufficient for agreement assessment.

### Limitations and Future Work

Several limitations should be addressed in future iterations. First, the composite verdict thresholds are based on established statistical conventions rather than calibrated against clinically meaningful differences, and a key next step is to align them with minimal important differences through expert panels and simulation. Second, although a patient-level random effect was added and admission year was used to confirm the absence of temporal drift in suppression, the random-intercept structure does not capture whether anonymization affects specific diagnosis groups differentially; random slopes or use cases with inferential targets less directly exposed to variance depletion would extend the analysis. Third, microaggregation was performed using the geometric mean so that within-cluster aggregation is consistent with the logarithmic scale on which LOS is modeled, which was used to reduce the positive bias that raw-scale arithmetic aggregation would introduce under a concave transformation. We acknowledge that a minor persistent bias may remain because geometric-mean microaggregation aligns with log(LOS), whereas the mixed model was fitted to log(LOS+1), creating a slight scale mismatch particularly at the lowest boundary where the LOS equals 1. A further residual limitation remains that microaggregation of any form compresses within-cluster variance, so mixed models fitted to microaggregated, variance-depleted outcomes may produce near-singular fits and overconfident inference. The compressed residual variance, sharply peaked density plots, very small standard errors, extreme *P* values, and strongly negative AIC values in the anonymized datasets should therefore be interpreted as signs that uncertainty estimates from anonymized data may represent lower bounds rather than fully reliable measures of precision. Fourth, the masking of records is nonrandom and concentrated on rare and longer-stay encounters, so estimates for those specific conditions warrant caution; pattern-mixture or tipping-point sensitivity analyses following Leurent et al [61], in the spirit of the missing-not-at-random framework of Little and Rubin [62], would test the robustness of the rank-concordance and magnitude results to worst-case assumptions about the masked encounters. Fifth, testing only 3 k values constrains the identification of inflection points, and including lower and higher thresholds, as well as further privacy models such as differential privacy, t-closeness, and l-diversity, would refine the dose-response characterization. Sixth, the evaluation used a single tool, ARX, under one configuration family, so the concept should be understood as a software-agnostic approach illustrated through a single-tool case study; a multitool comparison would substantiate the generalizability claim. Seventh, and most importantly for external validity, the retained dataset deliberately includes only 2 dimensions, primary ICD code and LOS, and excludes variables such as age, sex, and admission and discharge dates. A 2D dataset of this kind has limited standalone utility for epidemiological analyses, patient care-pathway studies, or key-performance-indicator computation even before anonymization, and the saturation of LOS distortion at k=5 is a property of this low-dimensional configuration. Applying k-anonymity to a higher-dimensional release with additional quasi-identifiers would be expected to require more suppression and produce greater utility loss, so the saturation observed here should not be generalized to such settings. Eighth, LOS served simultaneously as a quasi-identifier and as the inferential outcome, a worst-case design that makes part of the observed inferential degradation an upper bound on what analyses unrelated to the transformed quasi-identifiers would experience.

### Recommendations

Medical informatics researchers, data integration centers, and data-protection officers should empirically verify the distributional and inferential fitness-for-purpose of any anonymized clinical dataset before secondary use. Before conducting analyses or sharing data, practitioners should apply distributional screening, including quantile-level characterization and variance diagnostics, to quantify the transformation footprint. Inferential reproducibility should then be tested for the specific association under investigation, using both rank-order and magnitude-sensitive agreement measures with CIs. Results from anonymized data should be reported alongside explicit documentation of the k-anonymity level, the complete anonymization configuration (including the transformation model applied to each quasi-identifier, suppression limits, optimization weights, and attacker models), the suppression rate and its distribution across quasi-identifiers and subgroups, the distributional impact metrics, and a statement on whether ICC inflation was observed and how it was interpreted (Textbox 1 and Checklist 1).

Textbox 1. **Suggested reporting-statement template for studies using anonymized clinical data.**

The analysis used data anonymized with [tool name, version] under [privacy model], applying [parameter setting(s)]. Quasi-identifiers were transformed as follows: [variable group 1: method], [variable group 2: method]. The resulting suppression rate was [X]%, distributed [uniformly/non-uniformly] across [subgroups / time]. The impact of anonymization on data utility was quantified with metrics selected to capture distributional preservation, structural or category preservation, and downstream inferential reproducibility, yielding [metric 1] = [X], [metric 2] = [X], and [metric 3] = [X] with 95% confidence intervals. The [agreement metric] changed from [X] in the original dataset to [X] after anonymization. Any apparent increase in agreement should be interpreted cautiously, as it may reflect variance deflation or structural simplification rather than genuinely improved analytical signal. The anonymized data may therefore be less suitable for analyses requiring high-fidelity variance structure, accurate absolute estimates, complete representation of sparse categories, or full preservation of original data granularity. Where the analytical target variable was itself subject to anonymization transformation, this was stated, and its amplifying effect on utility loss was noted.

To support consistent reporting, we created the ADAQI checklist (Anonymization and Data Analysis Quality and Impact Reporting; Checklist 1), a structured aid of 29 items organized across 7 reporting domains, and we propose that future publications use it for standardized reporting of anonymization impact. We provide a blank template in Checklist 1 and a worked, filled-in version completed against this study as a concrete example. Completion can be confirmed with a single sentence in the Methods section, for example, anonymization impact was reported using the ADAQI checklist (Checklists 1 and 2).

### Conclusions

In summary, this methodological case study demonstrates, using the ARX tool, a procedure that combines distributional fidelity assessment with inferential reproducibility testing for anonymized clinical data. Applied to 719,387 inpatient encounters, k-anonymity at k=5, 10, and 15 produced a measurable distributional change in LOS (KS *D*=0.147, SD compression of about 6.5 days, contraction of the upper tail) and progressive vocabulary erosion (Jaccard declining from 0.624 to 0.421), while preserving the rank ordering of diagnosis-associated LOS effects (Spearman ρ 0.964-0.970) and the aggregate magnitude agreement (Lin CCC 0.959-0.966). Within this preserved aggregate, a minority of low-signal diagnoses underwent extreme individual distortion, including sign reversals in approximately 7% of categories. None of these effects were signaled by the internal loss metric of the tool, which reported aggregate losses below 10%. The numeric distortion saturated at k=5, while categorical erosion accumulated with k. These findings are specific to a deliberately low-dimensional, 2-quasi-identifier worst-case design, and should not be generalized to higher-dimensional releases. The practical conclusion is that anonymized datasets of this type can support ordinal and comparative analyses of the diagnosis-to-LOS relationship, but can mislead analyses that depend on faithful variance structure, accurate absolute estimates, or complete diagnostic vocabulary. Furthermore, the analytical footprint of anonymization should be measured and reported rather than assumed. The open-source R implementation, the anonymization certificates, and the reporting checklist are available to enable independent replication. Multitool and multidataset validation constitute the next steps of the project.

## Supplementary material

10.2196/97661 Multimedia Appendix 1 R Project.

10.2196/97661 Multimedia Appendix 2 ARX certificate for k-anonymized data with k=5.

10.2196/97661 Multimedia Appendix 3 ARX certificate for k-anonymized data with k=10.

10.2196/97661 Multimedia Appendix 4 ARX certificate for k-anonymized data with k=15.

10.2196/97661 Multimedia Appendix 5 Detailed plots and tables.

10.2196/97661 Checklist 1 ADAQI checklist template.

10.2196/97661 Checklist 2 ADAQI checklist filled out.

## Abbreviations

ADAQI: Anonymization and Data Analysis Quality and Impact Reporting
AIC: Akaike information criterion
BLUP: best linear unbiased prediction
CCC: concordance correlation coefficient
ICC: intraclass correlation coefficient
ICD: International Classification of Diseases
ICD-10-GM: International Classification of Diseases, 10th Revision, German Modification
ICD-3: Three-character ICD category
KS: Kolmogorov-Smirnov
LMM: linear mixed model
LOS: length of stay
RMSE: root mean square error
RQ: research question
