# Supplementary material for: Quantifying the Impact of Anonymization-Induced Clinical Data Quality Loss: Methodological Quantitative Case Study Using Primary Diagnosis Codes and Hospital Length of Stay
Source: JMIR Med Inform. 2026 Sep 11;14:e97661. doi: 10.2196/97661 (PMC13566071; doi:10.2196/97661)
Supplement: Supplementary file 1 [file medinform-v14-e97661-s002.pdf]

Project

1. Properties

- Project
  - » Name: anon impact
  - » Description: Not specified

Input specification

1. Input data

- Data
  - » Records: 719387
  - » Attributes: 5

2. Attributes and transformations

- Insensitive attributes
  - » patient*id: Integer*
  - » admission*year: Integer*
  - » encounter*id: Integer*
- Sensitive attributes
  - » None
- Identifying attributes
  - » None
- Quasi-identifying attributes
  - » main*diagnosis*icd: String
  - » los*days: Integer*
- Response variables
  - » main*diagnosis*icd: String
  - » los*days: Integer*
- Generalization hierarchy
  - » Attribute: main*diagnosis*icd
  - » Height: 1
  - » Minimum level: 0
  - » Maximum level: 0
- Microaggregation function
  - » Attribute: los*days*
  - » Type: Geometric mean
  - » Clustering: No

3. Configuration

- Weights
  - » main*diagnosis*icd: 0.5
  - » los*days: 0.5*
- Settings
  - » Assume monotonicity: No
  - » Suppression limit: 0.5
- Risk thresholds
  - » Prosecutor risk: 0.2
  - » Journalist risk: 0.2
  - » Marketer risk: 0.2

ARX

Output properties

1. Output data

- Data
  - » Records: 719387
  - » Attributes: 5
- Checksum
  - » SHA-256: af13369be95a90ceb7ed31c2a20b57aa6edf318975bd3dadcc3cd9ec7ecb8082

2. Solutions

- Search space
  - » Size: 1
  - » Materialized: 1

3. Transformation

- Transformation
  - » Anonymity: Yes
  - » Minimum information loss: 0.07444213533300159
  - » Maximum information loss: 0.07444213533300159
    - Generalization scheme
      - » main*diagnosis*icd: 0/0

4. Data quality model

- Loss
  - » Aggregate function: Geometric mean
  - » Monotonic: No
  - » Generalization factor: 1.0
  - » Suppression factor: 1.0

5. Privacy models

- k-Anonymity
  - » Threshold (k): 5

ARX
