# Supplementary material for: Quantifying the Impact of Anonymization-Induced Clinical Data Quality Loss: Methodological Quantitative Case Study Using Primary Diagnosis Codes and Hospital Length of Stay
Source: JMIR Med Inform. 2026 Sep 11;14:e97661. doi: 10.2196/97661 (PMC13566071; doi:10.2196/97661)
Supplement: Supplementary file 4 [file medinform-v14-e97661-s006.pdf]

ADAQI Checklist

Anonymization & Data Analysis Quality and Impact Reporting

Version 1.0 · 7 sections · 29 items

Purpose

This checklist provides a systematic aid for reporting the design, execution, and analytical impact of data anonymization procedures in health and clinical research. It is structured to reflect the Methods, Results, Discussion flow of a research manuscript and is intended for use in methods sections, supplementary materials, or data availability statements.

Mandatory items (M) must be reported in all publications using anonymized data. Recommended items (R) should be reported wherever feasible. Conditional items (C) apply only when the described situation is present and must be reported if so.

M	Mandatory: must be reported	R	Recommended: report where possible	C	Conditional: report if applicable

Item	Reporting element	What to report	Done ☐
**A Data source & study design** 3 items · 2 mandatory			
A1	**Source dataset**	Dataset type (EHR, registry, claims…), institution, observation period, total number of records/encounters.	☐
A2	**Analytical purpose**	Intended use of the data: e.g., regression modelling, survival analysis, descriptive statistics.	☐
A3	**Regulatory/ethical context**	Applicable data - protection framework (GDPR, HIPAA, national law) and ethics approval number if available.	☐
**B Anonymization procedure** 6 items · 4 mandatory			
B1	**Tool name & version**	Anonymization software and exact version (e.g., ARX 3.9.2, sdcMicro 5.7.0).	☐
B2	**Privacy model**	Model applied: k - anonymity, ℓ - diversity, t - closeness, differential privacy, or a combination.	☐
B3	**Privacy parameter(s)**	All parameter values: k, ℓ, t, ε, δ. Report all tested levels if multiple were evaluated.	☐
B4	**Quasi - identifier specification**	List all quasi - identifiers, grouped by type (demographic, temporal, clinical), with the transformation method for each group (generalisation, suppression, microaggregation…).	☐
B5	**Sensitive attributes**	Variables designated as sensitive attributes and how they were protected (kept, suppressed, ℓ - diversified).	☐

B6	**Analytical target variable transformation**	If the outcome/target variable was itself transformed, describe the method and flag potential amplification of utility loss.	☐

C Suppression & data loss 4 items · 1 mandatory

C1	**Overall suppression rate**	Total proportion of records removed (n removed / N total × 100%). Report absolute n and percentage.	☐
C2	**Suppression rate by quasi - identifier**	Per - variable suppression rates (e.g., age: 3.2%, primary diagnosis: 8.7%).	☐
C3	**Suppression by patient subgroup**	Rates for clinically or demographically defined subgroups where suppression may be disproportionate (e.g., rare diagnoses, paediatric patients).	☐
C4	**Sparse - category loss**	If categories with <5–10 records were fully suppressed, describe their clinical or analytical relevance.	☐

D Distributional utility metrics 4 items · 4 mandatory

D1	**Metric selection rationale**	Justify the choice of metrics: at minimum one for distributional fidelity, one for categorical/structural preservation, and one for inferential reproducibility.	☐
D2	**Distributional fidelity metric**	Report a continuous distribution comparison metric (KS statistic D, Wasserstein distance, Earth Mover's Distance) with pre - and post - anonymization values.	☐
D3	**Categorical preservation metric**	Report a categorical association metric (Cramér's V, Jaccard similarity index) with pre - and post - anonymization values.	☐
D4	**Variance structure change**	Report changes in variance (SD, IQR) for key continuous variables. Flag variance deflation explicitly (e.g., microaggregation paradox: large SD compression with negligible mean shift).	☐

E Inferential reproducibility 5 items · 3 mandatory

E1	**Downstream model specification**	Describe the statistical model used to assess reproducibility (e.g., LMM with ICD - 3 random intercepts, Cox proportional hazards, logistic regression).	☐
E2	**Agreement metric before/after**	Report the chosen agreement metric (ICC, concordance correlation coefficient, BLUP concordance) on both original and anonymized data.	☐
E3	**Interpretation caveat**	If agreement or model fit appears to improve after anonymization, state explicitly that this may reflect variance deflation or structural simplification rather than a genuine improvement in analytical signal.	☐

E4	**Effect estimate comparison**	Compare key regression coefficients, odds ratios, or hazard ratios between original and anonymized datasets, including 95% confidence intervals.	☐
E5	**Random - effects structure change**	For multilevel models, report changes in random - effect variance components (ICC, between - cluster variance) pre - and post - anonymization.	☐

F Limitations & fitness - for - purpose 5 items · 2 mandatory

F1	**Analytical use - case limitations**	State explicitly which analytical purposes are impaired: high - fidelity variance modelling, accurate absolute estimates, rare - category analyses, granular longitudinal analysis.	☐
F2	**Generalisability statement**	Clarify whether findings from the anonymized dataset can be extrapolated to non - anonymized data or to other privacy parameter levels.	☐
F3	**Sensitivity analysis across privacy levels**	If multiple k (or ε) values were tested, report how utility metrics change across levels and identify the utility–privacy trade - off point.	☐
F4	**Comparison to published benchmarks**	Compare utility metric values to those reported in analogous studies using comparable data types or anonymization methods.	☐
F5	**Federated/distributed learning note**	If the data are used in a federated or distributed setting, describe how anonymization interacts with federation (e.g., privacy amplification, noise accumulation across sites).	☐

G Reproducibility & transparency 2 items · 1 mandatory

G1	**Code/configuration availability**	State whether the anonymization configuration (QI specification, tool settings) and analysis scripts are publicly available (e.g., GitHub URL). If not, state the reason.	☐
G2	**Anonymized data availability**	State whether the anonymized dataset is available (repository, upon request, not available) and under what conditions.	☐

Recommended reporting template

The following template may be adapted for the methods or results section of a manuscript. Bracketed placeholders must be replaced with study - specific values.

> *"The analysis used data anonymized with [tool, version] under [privacy model], applying [parameter(s)]. Quasi - identifiers were transformed as follows: [variable group 1: method], [variable group 2: method]. The suppression rate was [X]%. Anonymization impact was quantified via [metric 1 (distributional)] = [value], [metric 2 (categorical)] = [value], and [metric 3 (inferential)] = [value]. [Agreement metric] changed from [X] in the original dataset to [X] after anonymization. Any apparent increase in agreement should be interpreted cautiously, as it may reflect variance deflation or structural simplification rather than a genuine improvement in analytical signal."*

Note: Any apparent improvement in an agreement or reproducibility metric following anonymization should not be interpreted at face value. Variance deflation induced by generalisation or microaggregation can artificially narrow residual distributions and elevate agreement statistics without any genuine enhancement of analytical signal.

Notes on use

Scope. ADAQI applies to any dataset in which a formal privacy - preserving transformation has been applied prior to analysis, including k - anonymity, ℓ - diversity, t - closeness, and differential privacy mechanisms.

Adaptation. Items may be renumbered or merged in the manuscript. Authors are encouraged to cite the checklist and note any items not applicable to their study design.

Citation. When using this checklist, include the following statement in the methods section: "Anonymization impact was reported in accordance with the ADAQI checklist (v1.0)."
