# Supplementary material for: Quantifying the Impact of Anonymization-Induced Clinical Data Quality Loss: Methodological Quantitative Case Study Using Primary Diagnosis Codes and Hospital Length of Stay
Source: JMIR Med Inform. 2026 Sep 11;14:e97661. doi: 10.2196/97661 (PMC13566071; doi:10.2196/97661)
Supplement: Supplementary file 5 [file medinform-v14-e97661-s007.pdf]

ADAQI Checklist - Completed Example

Anonymization & Data Analysis Quality and Impact Reporting

Version 1.0 · 7 sections · 29 items

Worked, filled-in example completed against the study "Quantifying the Impact of Anonymization-Induced Clinical Data Quality Loss: A Methodological Case Study Using Primary Diagnosis Codes and Hospital Length of Stay." Mandatory (M) items are reported in all publications using anonymized data; Recommended (R) where feasible; Conditional (C) when applicable. A blank template is provided separately.

M	Mandatory: must be reported	R	Recommended: report where possible	C	Conditional: report if applicable

Item	Reporting element	Response (this study)	Type
**A Data source & study design** 3 items · 2 mandatory			
A1 M	**Source dataset**	EHR inpatient encounters from the clinical data warehouse of the Medical Data Integration Center, University Hospital Mannheim (UMM). Observation period 1 January 2010 to 30 September 2024. Total 719,387 encounters from 370,548 distinct patients.	☑
A2 M	**Analytical purpose**	Variance decomposition of log-transformed hospital length of stay (log(LOS+1)) by primary diagnosis using a three-level linear mixed model, plus distributional comparison of LOS and ICD-10-GM code distributions before and after anonymization.	☑
A3 C	**Regulatory/ethical context**	GDPR. Approved by Ethics Committee II, Medical Faculty Mannheim, Heidelberg University (ID 2024-885). Positively evaluated data-protection impact assessment and local Use and Access Committee approval.	☑
**B Anonymization procedure** 6 items · 4 mandatory			
B1 M	**Tool name & version**	ARX Data Anonymization Tool, version 3.9.2.	☑
B2 M	**Privacy model**	k-anonymity (record suppression combined with microaggregation).	☑
B3 M	**Privacy parameter(s)**	k = 5, 10, and 15, each applied to the same source dataset as a separate configuration. No l, t, epsilon, or delta (k-anonymity only).	☑
B4 M	**Quasi-identifier specification**	Two quasi-identifiers. Clinical: primary ICD-10-GM diagnosis code (categorical, no generalization hierarchy, height 1, resolved by cell suppression). Numeric: LOS in days (microaggregation by arithmetic mean). Equal optimization weights 0.50 each; geometric-mean loss; suppression limit 0.50; deterministic optimal search.	☑
B5 C	**Sensitive attributes**	No separate sensitive attribute was designated, as the model was k-anonymity rather than l-diversity or t-closeness. The patient identifier and admission year were retained outside the quasi-identifier set and not anonymized.	☑
B6 C	**Analytical target variable transformation**	Applicable and explicitly flagged. LOS served simultaneously as a microaggregated quasi-identifier and as the model response variable. This deliberate worst-case design amplifies utility loss, so the observed inferential degradation is reported as an upper bound.	☑
**C Suppression & data loss** 4 items · 1 mandatory			
C1 M	**Overall suppression rate**	No rows were deleted (0 encounters removed at every k). Suppression masked quasi-identifier cells with a missing token. Encounters with at least one masked cell: 5,564 (0.77%) at k=5, 12,315 (1.71%) at k=10, 18,858 (2.62%) at k=15.	☑
C2 R	**Suppression rate by quasi-identifier**	ICD and LOS were masked jointly in every affected encounter (record-level masking), so per-variable rates equal the encounter rates: ICD and LOS each 0.77 / 1.71 / 2.62% at k=5 / 10 / 15. Admission year was never suppressed (0%).	☑
C3 R	**Suppression by patient subgroup**	By admission year, masking was near-uniform (0.70-0.87% at k=5, 1.58-1.92% at k=10, 2.36-2.88% at k=15) with no temporal trend. By diagnosis, masking was non-random and concentrated on low-frequency codes (for example M85 43%, G82 33%, A31 78% of records at k=15). Masked encounters ran modestly longer (mean LOS about 7.2-7.5 vs 6.5 days; median 4 in both).	☑
C4 C	**Sparse-category loss**	Applicable. Suppression preferentially removed rare diagnoses. Estimable three-character ICD categories fell from 1,404 to 1,170 (k=5), 1,059 (k=10), 979 (k=15), so 234, 345, and 425 categories were no longer estimable. Clinical relevance (rare-disease erosion, planning bias) discussed in the manuscript.	☑
**D Distributional utility metrics** 4 items · 4 mandatory			
D1 M	**Metric selection rationale**	One metric per dimension: distributional fidelity (Kolmogorov-Smirnov D), categorical/structural preservation (Jaccard similarity and Cramer V), and inferential reproducibility (ICC and BLUP concordance via Spearman rho and Lin CCC).	☑
D2 M	**Distributional fidelity metric**	Two-sample KS D for LOS, as a descriptive divergence measure. D = 0.147 at all three k levels (scaled z about 87.6-87.8). The near-constant value indicates the distributional shift	☑

		saturates at k=5.	
**D3 M**	**Categorical preservation metric**	ICD-10-GM Jaccard similarity fell from 0.624 (k=5) to 0.493 (k=10) to 0.421 (k=15). Cramer V rose from 0.062 to 0.093 to 0.114. Pre-anonymization reference: Jaccard 1.0, no frequency redistribution.	☑
**D4 M**	**Variance structure change**	Microaggregation paradox explicitly flagged. LOS median shifted by one day and IQR narrowed from 5 to 4 days, while the standard deviation compressed by about 6.5 days (mean shift about -1.81 days, SD shift about -6.45 days). Large SD compression with a small location shift signals variance deflation.	☑
**E Inferential reproducibility**	5 items · 3 mandatory		
**E1 M**	**Downstream model specification**	Three-level linear mixed model, log(LOS + 1) with random intercepts for ICD-3 category and patient, admission year retained for a temporal-drift check. Estimated by REML (lme4 1.1-35.5).	☑
**E2 M**	**Agreement metric before/after**	Diagnosis ICC rose from 0.294 (original) to 0.827 / 0.831 / 0.837 at k=5 / 10 / 15. BLUP rank concordance (Spearman rho) 0.970 / 0.966 / 0.964. BLUP magnitude agreement (Lin CCC) 0.966 / 0.965 / 0.959. Confidence intervals reported in the manuscript (Table 3).	☑
**E3 M**	**Interpretation caveat**	Stated explicitly. The rise in ICC and conditional R-squared after anonymization reflects residual-variance deflation and structural simplification (removal of small diagnosis groups), not a genuine improvement in analytical signal. Marginal R-squared was near zero in every dataset.	☑
**E4 R**	**Effect estimate comparison**	Per-diagnosis BLUP effects compared between original and anonymized models. Aggregate magnitude agreement was high (Lin CCC 0.959-0.966; bias-correction factor Cb about 0.995), yet approximately 7% of low-signal diagnosis categories showed sign reversals, concentrated among diagnoses with original effects near zero (for example C61, C30, C31). Median BLUP ratio about 1.04-1.05; BLUP-pair MAD 0.077-0.084.	☑
**E5 C**	**Random-effects structure change**	Applicable (multilevel model). Between-diagnosis variance rose from 0.200 to 0.231 / 0.228 / 0.234; between-patient variance fell from 0.081 to about 0.010; residual variance collapsed roughly tenfold from 0.398 to about 0.036-0.038. Combined ICC (diagnosis + patient) rose from 0.414 to 0.864-0.873.	☑
**F Limitations & fitness-for-purpose**	5 items · 2 mandatory		
**F1 M**	**Analytical use-case limitations**	Impaired uses stated: high-fidelity variance modelling, accurate absolute LOS estimates, rare-category and rare-disease analyses, and any analysis depending on faithful distributional shape. Ordinal and comparative (rank-order) analyses of the diagnosis-to-LOS relationship remain feasible with caution.	☑
**F2 M**	**Generalisability statement**	Findings are specific to a deliberately low-dimensional, two-quasi-identifier worst-case design and should not be generalized to higher-dimensional releases, where additional quasi-identifiers would be expected to require more suppression and produce greater utility loss.	☑
**F3 R**	**Sensitivity analysis across privacy levels**	Three k values (5, 10, 15) were tested. LOS distortion and inferential metrics saturate at k=5 (step-function), while categorical vocabulary erosion accumulates with k (proportional gradient). The dominant utility cost is incurred at the first anonymization step.	☑
**F4 C**	**Comparison to published benchmarks**	Results contextualized against Pilgram et al, Johann et al, Jakob et al, Ohno-Machado et al, Cohen et al (2026), and Kaabachi et al (2025), all reporting task-dependent utility degradation under privacy-preserving transformation.	☑
**F5 C**	**Federated/distributed learning note**	Not applicable. The analysis was performed on a single-centre dataset and did not involve a federated or distributed-learning setting.	☑
**G Reproducibility & transparency**	2 items · 1 mandatory		
**G1 M**	**Code/configuration availability**	Publicly available under the MIT License on GitHub. The repository includes the R implementation of the impact function, the full analysis pipeline, the quasi-identifier specification, and the ARX anonymization certificates for k=5, 10, and 15 (Multimedia Appendices 1-5).	☑
**G2 R**	**Anonymized data availability**	Raw and anonymized data cannot be shared publicly due to data-protection requirements. Source data are archived and access can be granted for individual requests subject to Ethics and Use and Access Committee approval.	☑

Completed reporting statement (this study)

"The analysis used data anonymized with the ARX Data Anonymization Tool, version 3.9.2, under k-anonymity, applying k = 5, 10, and 15. Quasi-identifiers were transformed as follows: primary ICD-10-GM diagnosis code by cell suppression (no generalization hierarchy), hospital length of stay by microaggregation (geometric mean), with equal 0.50 weights and a geometric-mean loss function. No rows were deleted; the suppression rate (encounters with a masked cell) was 0.77% at k=5, 1.71% at k=10, and 2.62% at k=15. Anonymization impact was quantified via KS D (distributional) = 0.147, Jaccard (categorical) = 0.624 to 0.421, and BLUP concordance (inferential, Lin CCC) = 0.966 to 0.959. The diagnosis ICC changed from 0.294 in the original dataset to 0.827-0.837 after anonymization. This apparent increase in agreement should be interpreted cautiously, as it reflects variance deflation and structural simplification rather than a genuine improvement in analytical signal."

Citation. Anonymization impact was reported in accordance with the ADAQI checklist (v1.0).
