# Supplementary material for: Quantifying the Impact of Anonymization-Induced Clinical Data Quality Loss: Methodological Quantitative Case Study Using Primary Diagnosis Codes and Hospital Length of Stay
Source: JMIR Med Inform. 2026 Sep 11;14:e97661. doi: 10.2196/97661 (PMC13566071; doi:10.2196/97661)
Supplement: Supplementary file 7 [file medinform-v14-e97661-s005.zip › Multimedia Appendix 5 - Detailed plots and tables/Table1_cohort_characteristics.docx]

Table 1. Cohort Characteristics of Hospital Encounter Data

| Characteristic | Value |
| --- | --- |
| **Study period** | **2010-01-01 – 2024-09-30** |
| **Total hospital encounters, n** | **719,387** |
| Unique encounters, n | 719,387 |
| Unique patients, n | 370,548 |
|  |  |
| **Patient-level encounter structure** |  |
| Patients with one encounter, n | 239,373 |
| Patients with more than one encounter, n | 131,175 |
| Encounters from patients with more than one encounter, n | 480,014 |
| Maximum encounters per patient, n | 184 |
|  |  |
| **Encounters per year** |  |
| Min | 34,838 |
| Max | 60,616 |
| Mean (SD) | 47,959.1 (6,501.4) |
| Median | 47,720 |
|  |  |
| **Length of stay (days) – Overall** |  |
| Min | 1 |
| Max | 557 |
| Mean (SD) | 6.50 (9.68) |
| Median (IQR) | 4 (5) |
|  |  |
| **Top 5 principal diagnoses (ICD-10-GM)** |  |
| Z38.0 | 16,971 (2.36%) – Mean annual prevalence: 2.50% (±1.33%) |
| G47.31 | 14,020 (1.95%) – Mean annual prevalence: 1.92% (±0.44%) |
| C61 | 11,207 (1.56%) – Mean annual prevalence: 1.51% (±0.86%) |
| S06.0 | 10,368 (1.44%) – Mean annual prevalence: 1.40% (±0.45%) |
| I63.4 | 7,941 (1.10%) – Mean annual prevalence: 1.11% (±0.17%) |
